# Investigating orientation adaptation following naturalistic film viewing

**DOI:** 10.1038/s41598-025-21383-x

**Published:** 2025-09-26

**Authors:** Emily J. A-Izzeddin, Reuben Rideaux, Jason B. Mattingley, William J. Harrison

**Affiliations:** 1 Department of Experimental Psychology, Justus Liebig University Giessen, Giessen, Germany; 2 Center for Mind, Brain and Behavior (CMBB), Universities of Marburg, Giessen, and Darmstadt, Marburg, Germany; 3 Queensland Brain Institute, The University of Queensland, St Lucia, QLD 4072 Australia; 4 School of Psychology, The University of Sydney, Camperdown, NSW 2006 Australia; 5 School of Psychology, The University of Queensland, St Lucia, QLD 4072 Australia; 6 School of Health, University of the Sunshine Coast, Sippy Downs, QLD 4556 Australia

**Keywords:** Visual adaptation, Naturalistic film viewing, Generalised linear multilevel modelling, Image filtering, Tilt-aftereffect, Human behaviour, Perception

## Abstract

Humans display marked changes to their perceptual experience of a stimulus following prolonged or repeated exposure to a preceding stimulus. A well-studied example of such perceptual adaptation is the tilt-aftereffect. Here, prolonged exposure to one orientation leads to a shift in the perception of subsequent orientations. Such a capacity to adapt suggests the tuning of the visual system can change over time in response to our current visual environment. However, it remains unclear to what extent adaptation occurs in response to statistical regularities of features present in naturalistic scenes, such as oriented contrast. We therefore investigated orientation adaptation in response to natural viewing of filtered live-action film stimuli. Within a session, participants freely viewed 45 min of a film which had been filtered to include increased contrast energy within a specified orientation band (0°, 45°, 90°, or 135°; i.e., the adaptor). To measure adaptation effects, the film was intermittently interrupted to have participants perform a simple orientation judgement task. Having participants complete behavioural trials throughout the testing session, including 45 min of total adaptation time, allowed investigation of the accumulation of response biases and changes in such biases over the course of the session. We found very little evidence of adaptation across our conditions. Indeed, in the very few conditions where significant adaptation was observed, these effects were much weaker than those observed under typical tilt-aftereffect paradigms. Further, within a single session, we observed inconsistent development of adaptation effects. The current findings therefore suggest very minimal and, where present, inconsistent effects of adaptation in response to naturalistic viewing conditions. The divergence of our results from those predicted by prior studies using minimalistic studies, and suggests consideration of further barriers to understanding perceptual adaptation as experienced in nature are needed.

**Supplementary Information:**

The online version contains supplementary material available at 10.1038/s41598-025-21383-x.

## Introduction

The key to any organism’s success is its ability to adapt to its environment. Humans, for example, are exposed to and interact with many different environments on a daily basis, behaviourally adapting to context-specific conditions with relative ease. This is no small feat, with the unique requirements of each environment ranging from complex factors that we are consciously aware of (e.g., understanding whether we need to change our clothing to better suit current weather conditions) all the way down to basic perceptual factors that we have little to no awareness of (e.g., determining how well the distribution of basic visual features, such as orientation, match our prior expectations)^1–4^. The extent to which each environment differs and our ability to cope with such variations suggests a flexible and adaptive mechanism underlying our interactions with our environment.

**Fig. 1:**
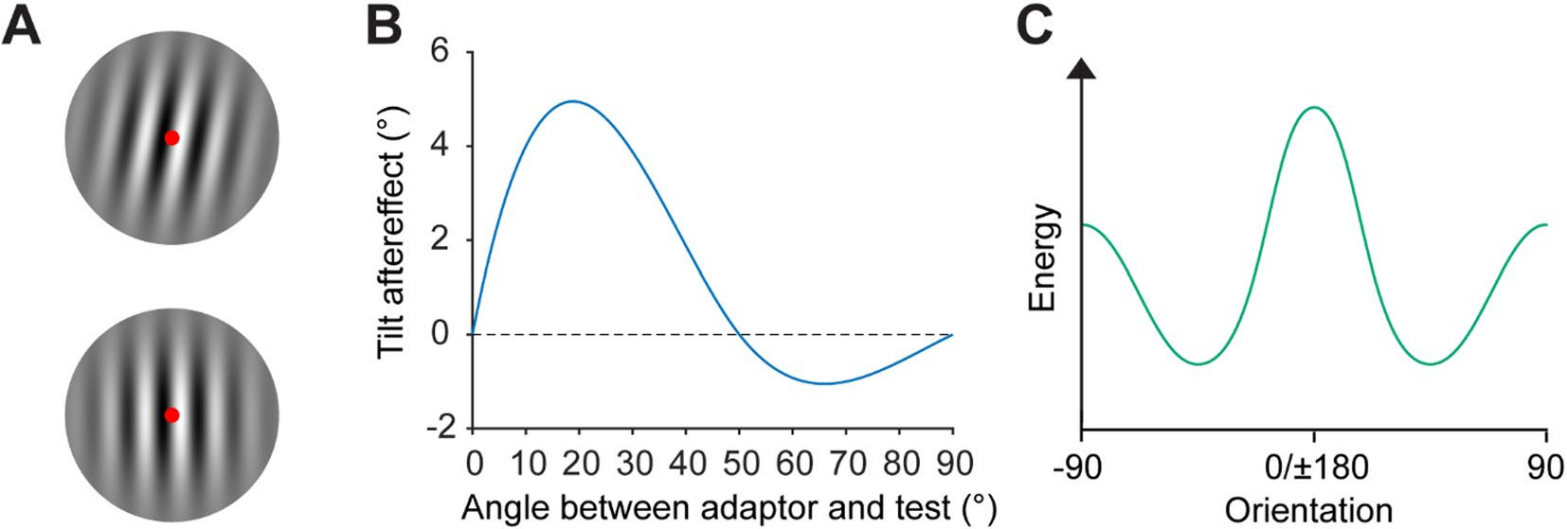
**Tilt aftereffect and orientation prior overview.** **(A)** Example of the tilt aftereffect. Stare at the central red fixation dot of the top Gabor patch for 30 s, then shift your fixation to the fixation dot of the bottom Gabor patch. The bottom patch may appear to be tilted counterclockwise despite being vertical. This is the tilt aftereffect, which is a perceived repulsion away from the orientation of the adapting Gabor. **(B)** Stereotypical aftereffects observed as a function of the difference in orientation between the adaptor and test stimulus. Positive values indicate a repulsive effect, and negative values indicate attraction. **(C)** Typical distribution of orientations observed in naturalistic images, with a dominance of cardinal orientations over obliques. This dominance has led to our prior for orientation information, resulting in the oblique effect. (adapted from Clifford et al., 2000)^8^.

At the level of visual perception, humans’ ability to adapt to specific visual features, such as orientation, has led to important insights into basic visuo-cognitive function. One of the most well-known approaches to understanding adaptation in this domain is the use of the classic “aftereffect” paradigm, initially described by Gibson and Radner^5^. Such paradigms are typically characterised by having participants fixate an oriented “adaptor” stimulus for several seconds. Subsequently, participants observe a “test” stimulus, positioned at a new orientation, and are asked to report the test stimulus’ orientation relative to a specified orientation (e.g., vertical). The hallmark finding in such paradigms is a perceptual “tilt-aftereffect”, occurring after the removal of the adaptor stimulus, whereby perception of a subsequent test stimulus’ orientation is altered relative to the adapted orientation (Fig. 1A-B)^5–11^. Specifically, if the test orientation is within ~ 50° of the adaptor orientation, a repulsive effect is observed, whereby the test stimulus appears to be oriented away from the adaptor orientation (Fig. 1B, **positive values**)^8^. Conversely, for test orientations greater than ~ 50° relative to the adaptor orientation, an attractive effect is observed, whereby the test stimulus appears to be oriented towards the adaptor orientation (Fig. 1B, **negative values**)^8^. The strength of such adaptation effects has been measured as a function of adaptation time, with results suggesting that the tilt-aftereffect builds up logarithmically in response to longer adaptation times^12–14^, saturating after approximately an hour^12,13^. Indeed, the fact that our perception is demonstrably impacted by an immediately preceding stimulus exemplifies our visual system’s capacity for highly specific short-lasting adaptation to its most recent input.

Biologically, the tilt-aftereffect is thought to arise from the adaptation of neurons involved with processing the adaptor stimulus, which undergo response suppression after prolonged or repeated stimulation^15^. This response suppression leads to overall shifts in neural preferential tuning, resulting in a distorted representation of the subsequent test orientation relative to the adaptor^15,16^. The fact that such tuning shifts occur has been suggested to be of functional benefit to the observer, acting to dynamically tune the visual system to optimally process the visual information yielded from the current visual environment^17,18^.

Beyond short-lasting adaptation to immediate sensory input, we possess broader heuristics that we use to interpret the elements of our environment – expectations, or priors, thought to be built up over the course of our lives^19–21^ and even over evolutionary timescales^22,23^. Of particular relevance to the tilt-aftereffect literature is our prior for orientation distributions. In nature, there is a dominance of cardinal orientations over oblique orientations (Fig. 1C)^24–29^, a pattern reflected in observers’ greater sensitivity to cardinal orientations than obliques, otherwise known as the “oblique effect”^23,26,30–39^. Indeed, the oblique effect has also been linked to well-documented perceptual biases, whereby observers experience repulsion of off-cardinal orientations away from the nearest cardinal^30,36,38,40,41^. These anisotropic biases have been observed under a multitude of experimental paradigms. However, the flexibility of the prior underlying such anisotropic biases within the constraints of an experimental session has received relatively less attention in the literature as compared with typical short-lasting adaptation effects.

Importantly, the probing of long-term priors for orientations is facilitated by the use of complex stimuli, such as naturalistic images. Complex stimuli allow for investigation of cross-channel interactions, which may be less obvious when defining simple stimuli a priori^42,43^. Indeed, naturalistic stimuli diverge from classic adaptation investigations that typically involve a single adaptor and no conflicting information, likely isolating response channels in a manner that is relatively different to natural feature processing^6,7,9,10^ (but see^44^. The relative lack of investigations of adaptation using complex stimuli therefore leaves open the question of whether, and at what timescales, naturalistic distributions of ordered spatial structure that invoke high-level object and scene representations elicit even low-level adaptation effects.

Beyond the use of naturalistic images, some studies have gone so far as to employ live-action films to better simulate naturalistic viewing conditions. For example, Dorr and Bex (2013) investigated the interplay between visual sensitivity and eye movements, having participants perform a detection task for a contrast increment target embedded in live-action films^45^. Similarly, Wallis et al. (2015) investigated luminance contrast sensitivity using a target detection paradigm, similarly embedded in a live-action film^46^. Of most relevance, Bex et al. (2009) investigated participants’ contrast sensitivity function after undergoing adaptation to live-action film stimuli^47^. Bex et al. found that, compared with when no adaptor stimulus was presented, contrast sensitivity was reduced for low spatial frequencies following live-action film adaptation. As such, Bex et al. demonstrate the capacity to elicit adaptation effects for contrast sensitivity under natural viewing conditions. However, the aforementioned investigations have focused on contrast sensitivity in conjunction with live-action film stimuli, leaving open the question of whether adapted orientation sensitivity can be elicited from live-action film.

**Fig. 2:**
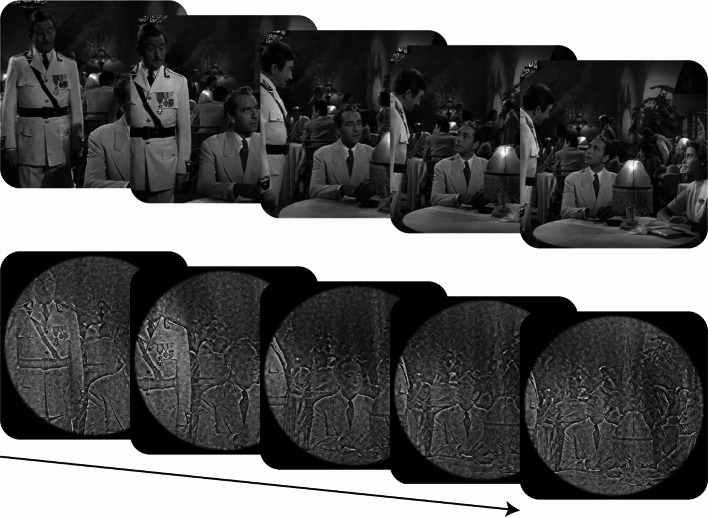
**Adaptation in response to naturalistic image stimuli.** In the current study, we presented participants with filtered film stimuli – in this case, from the film *Casablanca*. The top row are original film frames. Stimuli used in the experiment (bottom row) were filtered to only have one orientation present at relatively low spatial frequencies (1-4 cpd) – 0° in this case. Using this stimulus, we assessed participants orientation adaptation in response to naturalistic images. Note: spatial frequency filters were based on actual stimulus presentation size. Hence, examples here will be distorted by reducing the size of the images.

Therefore, in the current study, we investigated orientation adaptation by filtering live-action film stimuli. We filtered a film to have uniform orientation energy across all spatial frequencies, except for at relatively low spatial frequencies, which were filtered to contain only one specified orientation (i.e., the adaptor orientation; Fig. 2). Specifically, participants were shown the film in halves across separate days. Each film half (45 min of footage) was subjected to one of four filtering conditions, such that each participant saw 45 min of the film with only one cardinal orientation (i.e., 0° or 90°) present at relatively low spatial frequencies, and the other 45 min with only one oblique orientation (i.e., 45° or 135°). Differences between cardinal and oblique adaptors were of interest because the anisotropic encoding of orientations could yield differential adaptation effects^32,48–50^. The filtered film was intermittently interrupted to have participants perform a basic orientation judgement task, indicating whether a centrally presented test grating was rotated clockwise or counterclockwise relative to a peripheral bar. By having participants freely view a film stimulus, we presented naturalistic images in an engaging manner that more closely simulates participants’ real-world experience than typical tilt-aftereffect paradigms. Additionally, having participants view the film for 45 min over the course of a session allowed us to investigate the timeline of any adaptation that occurred. Therefore, the use of such complex stimuli in conjunction with a clockwise/counterclockwise orientation judgement task allowed us to measure participants’ degree of adaptation to orientations presented under more naturalistic viewing conditions over time.

## Methods

### Participants

Twenty-eight participants took part in the current study. Four participants were excluded – three failed the baseline data quality check (see Sect. Practice and baseline testing), and one participant withdrew. Hence, 24 participants’ datasets were included in analyses. Participants had varying degrees of experience participating in psychophysical experiments, and all participants were naïve to the purpose of the experiments. All participants provided informed consent to participate in the current study. Ethics approval was granted by The University of Queensland (Medicine), Low & Negligible Risk Ethics Sub-Committee, with all methods performed in accordance with the Declaration of Helsinki.

### Stimuli

#### Video stimuli

**Fig. 3:**
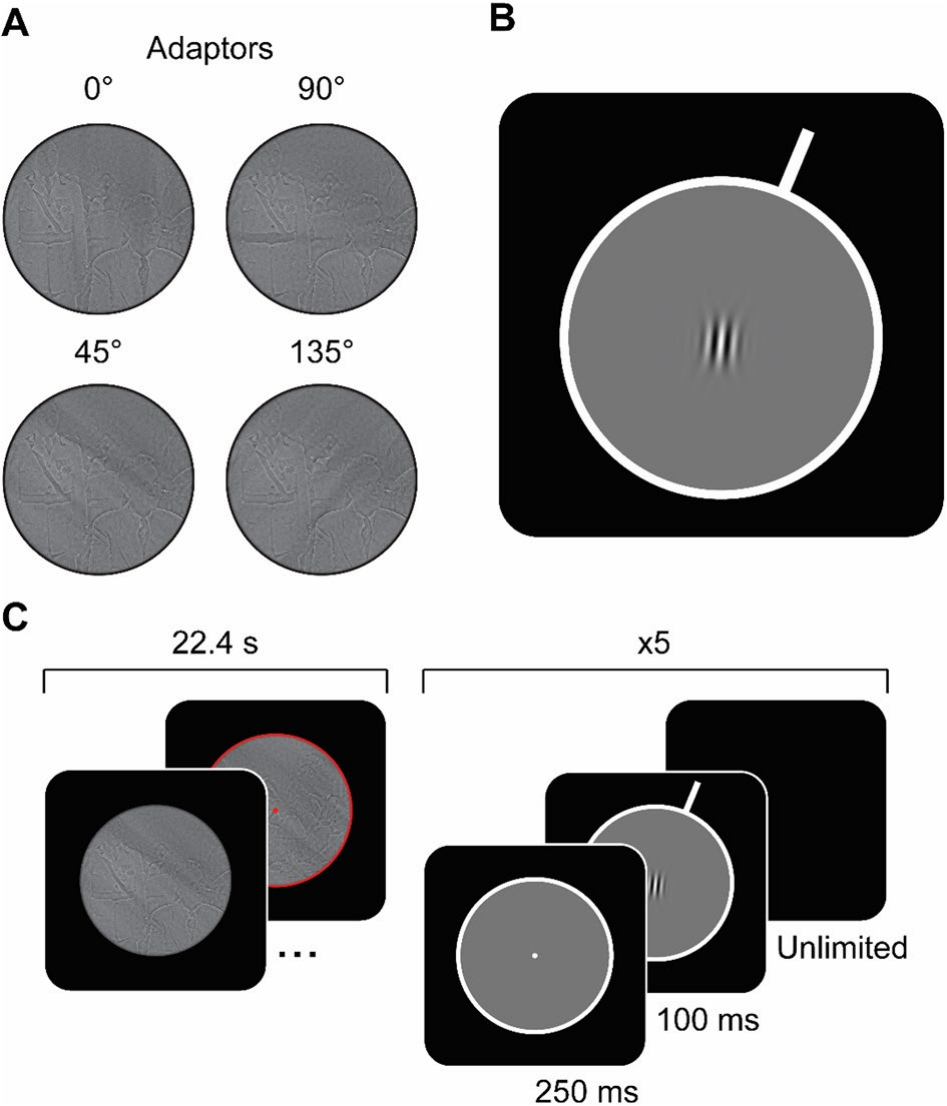
**Overview of stimuli. (A)** An example of the same frame from the film, *Casablanca*, after passing through one of four orientation/spatial frequency filters, resulting in all orientations at 1-4 cpd being removed, except for 0°, 90°, 45°, or 135°. Note: Images displayed here are smaller than those displayed to participants, hence filtering visible here will not correspond to 1–4 cpd. **(B)** Example of the perceptual task stimulus. Observers reported whether the peripheral white bar (the standard stimulus) was oriented clockwise or anti-clockwise relative to the central test grating. The white bar was positioned peripherally, outside of the area taken up by the adaptor stimulus. Because participants were viewing the video itself, the standard should not be subject to the same level of adaptation as the central test grating (which did occupy the same space as the preceding adaptor). **(C)** Participants viewed a 22.40 s filtered film clip, followed by five trials wherein participants were asked to indicate whether the central grating is tilted clockwise or counterclockwise relative to the standard stimulus (i.e., the protruding white bar). This structure repeated 120 times in each session until participants had watched 44.80 min of the film, *Casablanca*, and had completed 600 trials.

*Casablanca* was used as the source film for stimulus generation (the film is dedicated to the public domain under CC0)^51^. We extracted each frame from the digitised film as individual image files. Each frame was first whitened such that there was equal energy at all orientations and spatial frequencies. Subsequently, a 1/f spectrum was applied to the frames. We altered the frames to contain specified orientations at particular spatial frequencies by implementing standard image processing techniques. First, we take a given frame, convert it into the frequency domain, and whiten its amplitude spectrum. We then multiply the amplitude spectrum by a spatial frequency filter (leaving only the desired spatial frequencies) and an orientation filter (leaving only the desired orientations), and convert back into the spatial domain. This process results in a movie frame that includes contrast energy only within the desired orientation and spatial frequency channels^52^. The spatial frequency filter was flat-topped, uniformly covering 1-4 cpd, with cosine edges falling to zero over half an octave. The orientation filters were raised cosine filters with a periodicity of 45°. Four versions of the orientation filter were generated, such that four versions of the film were produced that had each frame filtered to contain only 0°, 45°, 90°, or 135° orientations (i.e., the possible adaptor orientations) at the specified spatial frequencies (Fig. 3A). Filtering at 1-4 cpd meant that filtered orientations were in a frequency range that approximately corresponds to the peak tuning of humans’ contrast sensitivity function^47^. Finally, each final frame’s total energy was matched to that of the original unfiltered frame, thereby retaining the overall energy dynamics of the original film. We then cropped frames to a 14.30°-diameter circle – the maximum possible diameter given the film frame size, display resolution, and viewing distance. We cropped frames using a circular aperture to remove cardinal orientation cues that would have otherwise been introduced at the edges of the stimulus.

After filtering, film frame images were re-combined to make a series of 22.40s video clips, generating a total of 240 clips that amounted to 89.6 min. We chose the current clip length to break up the film into enough segments to allow for fewer behavioural trials (i.e., five) between each clip, and to allow for an even number of clips in each session (i.e., 120; see Sect. Adaptation). We also included the associated audio files in generated clips to allow participants to listen to the film while watching.

At the end of a clip, participants immediately completed behavioural task trials. As such, we included a cue to warn participants that the clip was about to end, and a trial was about to start. The cues were a 0.12°-stroke red border that appeared around the edge of the stimulus, as well as a central 0.11° red fixation point. The cues were presented for the final second of each clip (Fig. 3C, left).

#### Perceptual task stimuli

Participants watched the filtered video clips in sequence, with a break between each clip to collect behavioural responses. All stimuli were presented on a black background, with a centrally positioned 14.30° grey circle, framed by a white border (stroke = 1.00°; Fig. 3B). The fixation point, when shown, was presented centrally, subtending 0.11°. The trial stimulus consisted of two components: the standard stimulus and the test stimulus. The standard stimulus was made up of one white bar, 2.24° in length and 1.00° stroke, extending from the white border surrounding the central grey circle at one of four orientations (−67.5°, −22.5°, 22.5°, or 67.5°). The test stimulus was defined in the frequency domain by a two-dimensional raised cosine function, with the radial axis corresponding to orientation and the tangential axis corresponding to spatial frequency. The full-width half height (i.e., bandwidth) of the filter was 45° for orientation, and 1.148 cpd for spatial frequency. Note that the resulting test stimulus’ spectrum was therefore well-equated with the profiles of the filters used to generate the adapting stimuli, defined by a spatial frequency filter uniformly covering 1-4 cpd, and an orientation filter with a bandwidth of 45°. The test stimulus was drawn centrally. Test gratings were presented at 100% RMS contrast (with recent papers demonstrating tilt aftereffects with high-contrast test stimuli)^53–55^. The grating had a spatial frequency of 2 cpd and a 0° phase. Test gratings on each trial were drawn at one of seven orientation offsets relative to the standard stimulus (−10°, −5°, −2.5°, 0°, 2.5°, 5°, or 10°).

### Apparatus

Stimuli were generated on a ThinkStation P520 computer (running Windows 10 Enterprise) with the Psychophysics Toolbox (3.0.17) for MATLAB (R2018a)^56,57^. Stimuli were presented on a 24-inch Asus VG428QE 3D monitor with 1920 × 1080-pixel resolution and a refresh rate of 100 Hz. A gamma correction was applied to the display, assuming a gamma of 2. As noted by Bex et al. (2005), small errors in gamma are inconsequential to the representativeness of the naturalistic images^58^. A cardboard circular mask was superimposed on the monitor to block out orientation cues conveyed by the edges of the screen. Film audio was provided through Sony WH-CH710N Wireless Noise Cancelling Headphones (connected via AUX cable) at participants’ desired volume. Testing sessions were completed in a testing booth with the lights off.

### Task design

The following sections will outline the different elements of the experiment design. For a flow chart summarising the key session, trial, and task elements of the experiment, please see **Supplementary Figure ****S1**.

#### Condition counterbalancing

Each participant watched one half of the film with a cardinal adaptor (0° or 90°) and the other half of the film with an oblique adaptor (45° or 135°; 45 min per half). Combinations and order of adaptors were counterbalanced across participants. Participants were shown only one cardinal and one oblique adaptor to maximise the adaptation time and trials for each condition. Counterbalancing condition assignment allowed us to investigate adaptation to all adaptor conditions.

A participant experienced two standard stimulus orientations, which were the same for both of the adaptor orientation conditions the participant completed. Because the strength of the tilt-aftereffect depends on the orientation of the test stimulus relative to the adaptor orientation (Fig. 1B), standard stimuli orientations were selected to be equidistant to both adaptors viewed across sessions^8^. For example, if a participant viewed a 0 and 135° adaptor across sessions, the two standard orientations were 67.5 and − 22.5° for both sessions. For each combination of standard orientations, one was ± 22.5° separated from the adaptor, and the other was separated by ± 67.5°. Given all possible orientations of the test grating relative to the standard were tested, the orientations of the grating relative to the adaptor orientations were also balanced across conditions. Therefore, selecting standard orientations to be equidistant from adaptors meant that only the orientation of the adaptor changed across sessions.

#### General trial structure

Participants completed five perceptual task trials between film segments. Participants were alerted to the impending start of a perceptual trial via the appearance of a red border and fixation point for the last second of the film clip (see Sect. Video stimuli). On each behavioural trial, participants indicated whether the central test stimulus was rotated clockwise or counterclockwise relative to the standard stimulus (Fig. 3C, **right**). Trials began with a central white fixation point, presented for 250 ms, after which the stimulus was shown, with the standard stimulus at its given orientation for that trial and the test stimulus at its given orientation offset relative to the standard. The standard/test stimulus was presented for 100 ms, followed by a blank screen which remained until a response was recorded. Participants were instructed to use the left or right arrow keys to make their response. During trials, participants were instructed to fixate on the test stimulus when presented. Critically, the standard stimulus was positioned to be outside of the space occupied by the film stimulus (i.e., the adapting region) to minimise the impact of adaptation on perception of the standard.

Participants were not subject to eye tracking and were free to move their eyes while watching the video stimuli. As such, the retinotopically-mapped adaptation region likely extended beyond the confines of the spatially-mapped film stimulus area. Nonetheless, on average, we expect participants to look towards the centre of the film stimulus area^59^. Therefore, the level of adaptation experienced in the retinotopic areas that correspond to the standard stimulus should be weaker than that experienced foveally. Further, the brevity of the test and standard stimulus presentation (100 ms) would pose difficulty in attempting to foveate both stimuli. Hence, such a tactic would likely lead to decrements in task performance. Given participants’ performance is assessed after the initial testing session (see Sect. Practice and baseline testing), it is unlikely such saccades can explain performance observed.

#### Practice and baseline testing (Session 1)

Prior to adaptation, in a separate initial session, participants completed 588 practice trials without viewing any film stimuli. For the first 84 trials, test stimulus offsets relative to the two standard orientations used were doubled (i.e., to −20°, −10°, −5°, 0°, 5°, 10° and 20°). The final 504 practice trials had offsets matching the general trial structure (i.e., −10°, −5°, −2.5°, 0°, 2.5°, 5°, or 10°). Participants received feedback in the form of a red or green (incorrect and correct, respectively) fixation point, presented centrally for 750 ms following response. Following feedback, the next trial would begin.

Practice trials were followed by 600 experimental trials to quantify participants’ baseline orientation biases as measured by the task. No feedback was given for baseline trials. Baseline trials had test stimulus offsets matching the general trial structure described above. Given the limits placed on trial numbers due to time constraints, and that smaller offsets were expected to yield noisier responses, we had participants complete more trials for smaller offsets than larger offsets in order to constrain the psychometric fits described below. Specifically, for each of the two standard stimulus orientations, there were 20 trials each for − 10° and 10° offsets, 40 trials each for − 5° and 5° offsets, and 60 trials each for − 2.5°, 0°, and 2.5° offsets.

After a participant completed the baseline trials, we conducted a data quality check to evaluate if their performance was above chance. The participant’s baseline data were fit with two generalised linear regression models using MATLAB’s “fitglm” function. The first model was an intercept-only model, and the second included intercept and slope parameters, using a logit link function (i.e., a psychometric function). The log likelihoods for each model were compared and participants “passed” the data quality check if the psychometric function was a significantly better fit (*p* <.05) than the intercept-only model. Specifically, we computed the likelihood ratio chi-square statistic by first doubling the difference in log likelihoods between the two models. We then calculated the chi-square cumulative distribution function at this difference value, subtracted from 1, and interpreted the resulting value as a *p*-value^60,61^.

#### Adaptation (Sessions 2–3)

Participants’ second and third sessions each started with a block of 210 practice trials. Practice trials had test stimulus offsets matching the general trial structure. For the first 14 trials (one trial per standard stimulus orientation and test stimulus offset combination), participants received feedback. For the last 196 trials, no feedback was given, and there was an even distribution of trials across standard stimulus orientation and test stimulus offset combinations (i.e., 14 trials per combination). Responses for the last 196 trials were included in participants’ baseline dataset for analyses.

Practice trials were followed by the adaptation component of the session. During each adaptation session, participants observed 120 filtered video clips (each 22.40s in length) taken from the film, *Casablanca*, intermingled with trials. Clips were shown in the order they are presented during the film, such that participants were able to follow the original narrative structure. Participants completed 600 trials in each adaptation session. Trials followed the general trial structure and were split into 120 blocks of five, with each block preceded by a video clip (Fig. 3C). In the first adaptation session, participants watched the first 44.80 min of *Casablanca* with either an oblique adaptor or cardinal adaptor applied. In the second session, participants viewed the second 44.80 min with the opposite adaptor-type to that used in the previous session (counterbalanced across participants). Participants viewed the video with audio played through headphones.

At the end of each block of five trials, participants were shown a countdown timer, which was a circle that gradually filled like a timer, and gave participants an opportunity to have a break if needed. If participants requested a break, the screen went black, and participants could re-start when ready. Participants were asked to only take breaks when necessary. No participants left the dark testing booth during their break, minimising their exposure to the statistics of the broader laboratory during their breaks.

At the end of the final session, participants had viewed 89.60 min of *Casablanca*. However, there were an additional ~ 10 min of the film that were not filtered. Participants were given the option to view this after completing the experiment, acting as a potential indicator that participants were invested in attending to the film. We note that in this time, unprompted, at least one participant indicated that they did *not* realise that the film they had watched during the experiment was altered. They reported that it was only after they had seen the unaltered final 10 min of the film that they realised the experiment stimulus was a modified film. This participant reported that they assumed the altered film’s appearance was due merely to the film’s age. More than one participant made a similar comment about not noticing the film had been altered but, unfortunately, this was unexpected and we did not formally record the reports or the exact number of participants who made such comments.

### Analyses

#### Generalised linear multilevel modelling

**Fig. 4:**
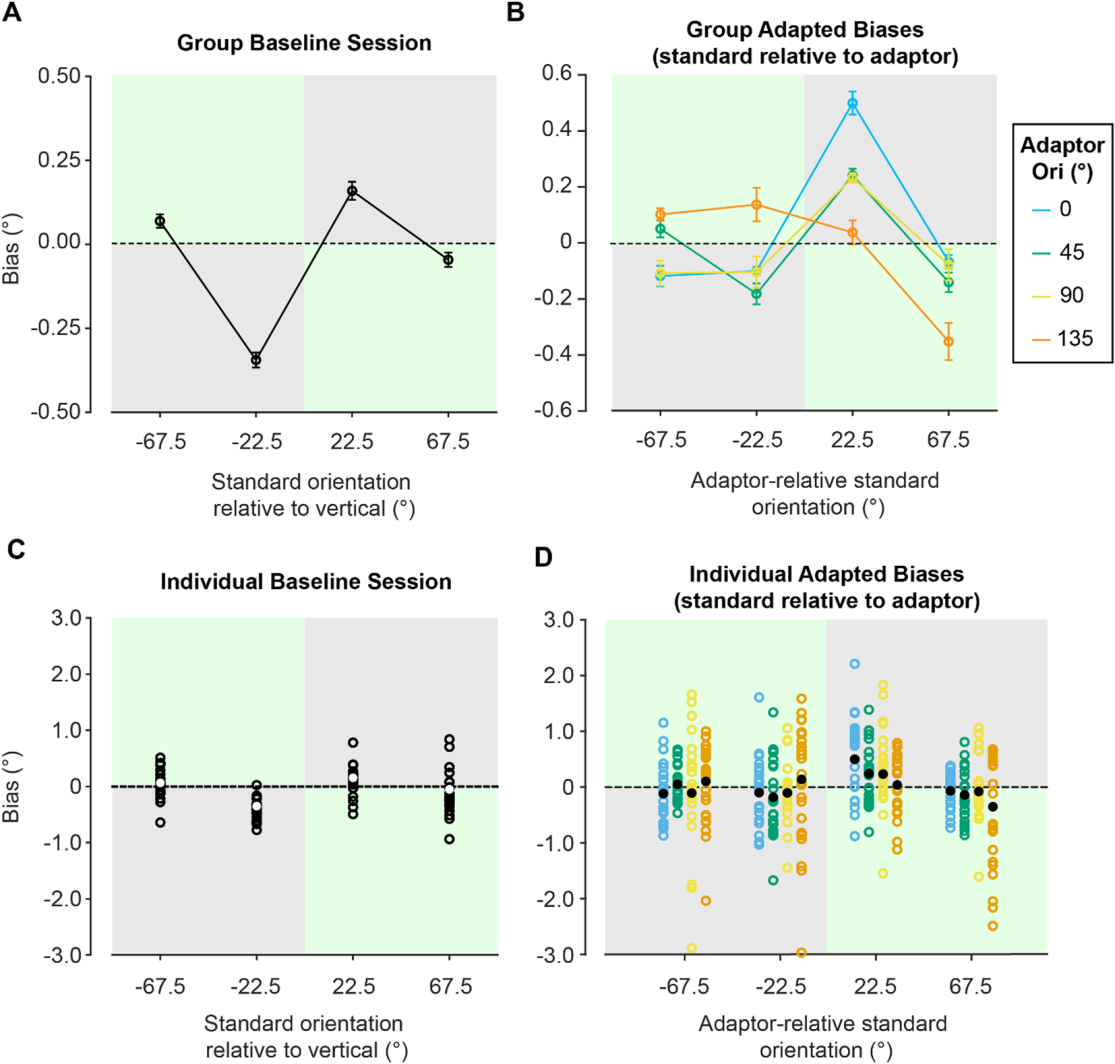
**Bias data from baseline and adaptation sessions. (A)** GLMM fits of participants’ baseline bias data with the standard orientation (relative to vertical) on the x-axis and bias shift on the y-axis. **(B)** GLMM fits of participants’ adaptation bias data relative to baseline for each adaptor orientation (see bottom legend). Here, standard orientations have been normalised to represent their orientation relative to the adaptor orientation. These data have had baseline data subtracted to view biases as a result of adaptation alone. Grey and green panels are used again to indicate repulsive and attractive biases, respectively. **(C)** Model fits for individual observers at each standard orientation (relative to vertical) for the baseline condition. White data points represent the overall beta weight estimate for each condition, as seen in Panel A. **(D)** Same as C, but for each adaptor orientation (different colours), at each standard orientation (normalised to be relative to the adaptor; x-axis). Here, overall beta weight estimates for each condition, as seen in Panel B, are shown in black.

Participants’ performance was modelled using a generalised linear multilevel model (GLMM) framework. The GLMM was fit to all of the data using MATLAB’s fitglme() function. Participant responses were coded as 0 and 1, indicating that the test stimulus was perceived to be oriented to the left or right of the standard, respectively. The model included four predictors based on trial-by-trial conditions. Specifically, the first predictor was the offset of the test stimulus relative to the standard (i.e., −10°, −5°, −2.5°, 0°, 2.5°, 5°, or 10°). The second predictor was the adaptor condition (i.e., 0°, 45°, 90°, or 135°). The third predictor was the standard orientation (i.e., −67.5°, −22.5°, 22.5°, or 67.5°). The fourth predictor was the interaction between adaptor and standard orientation conditions – implemented because the combination of standard orientations a given participant saw was dependent on the combination of adaptor conditions they observed across sessions. For the baseline condition, where no adaptor was used, an additional condition label of ‘−999’ was added to the adaptor condition predictor. All predictors were categorical. The probability of a right response was predicted as:

1 $$\:P\left(right\right)=probit\left(\eta\:\right)$$

where $\:probit$ represents the probit link-function, and:

2 $$\:\eta\:={\beta\:}_{0}+{\beta\:}_{1}T+{\beta\:}_{2}F+{\beta\:}_{3}S+{\beta\:}_{4}FS$$

where $\:{\beta\:}_{0}$ is the intercept term, $\:{\beta\:}_{1}$ is the weight of the test stimulus offset relative to the standard, $\:T$, $\:{\beta\:}_{2}$ is the weight of the adaptor condition, $\:F$, $\:{\beta\:}_{3}$ is the weight of the standard orientation, $\:S$, and $\:{\beta\:}_{4}$ is the weight of the adaptor/standard orientation interaction, $\:FS$. To partially pool coefficient estimates across participants, the GLMM included participant as a random effect. We describe this approach in more detail in Rideaux et al. (2022)^62^. For visualisation of fits (e.g., Fig. 4), we found the GLMM estimate for each adaptor by standard orientation condition combination, representing the conditional intercept, which we interpret as observers’ response bias for a given condition. To provide insight into the uncertainty surrounding the model’s estimates, we take the model’s trial by trial response predictions, find the average predicted response for the trials attributed to each individual observer within each condition. These average response prediction values were then be used to estimate the standard error of the model’s predictions within each condition.

#### Exploratory sequential analyses

Because we were interested in how adaptation effects emerge and evolve over prolonged viewing of systematically skewed image statistics, we conducted an exploratory sequential analysis to investigate changes in response bias across trials during testing sessions. Sequential analyses were conducted for each standard orientation tested. The possible standard orientations a participant saw were one of −22.5° and 22.5° and one of −67.5° and 67.5°. When participants make a left/right response relating to whether the test is rotated to the left/right of the standard, the response will be attractive or repulsive depending on the standard orientation. For example, if we consider whether a response is attractive relative to the nearest cardinal, a leftward bias for the − 22.5° standard orientation condition is attractive towards the nearest cardinal (i.e., vertical). However, a leftward bias for the 22.5° condition would be repulsive. However, this mapping of attractive vs. repulsive responses arising from adaptation will change depending on the orientation of the adaptor. Therefore, to allow for consistent interpretation, data were normalised such that standard orientations were re-coded to indicate their orientations relative to the adaptor orientation.

Response bias is equivalent to the average response made across trials. Hence, to measure changes in response bias for a single relative standard orientation, we calculated the cumulative average response with each new trial. For example, if participants responded with “right” on the first trial (coded as 1 for this analysis), then the response bias for the first trial will perfectly equal 1. Then, for the second trial, we take the average of this second trial as well as the first trial. Should participants respond “left” for the second trial (coded as −1 for this analysis), then the response bias will average out to 0. This process continues until the last trial in a given session/for a particular standard orientation, which calculates the average response across all trials (300 in total per session/standard orientation). Because there were equal numbers of trials where the target was offset to the left and right of the standard, deviations from 0 indicate an overall response bias. Put differently, should participants display no response bias across the session, the sequential analysis should quickly even out to roughly 0 and remain consistent across the sequence.

**Fig. 5:**
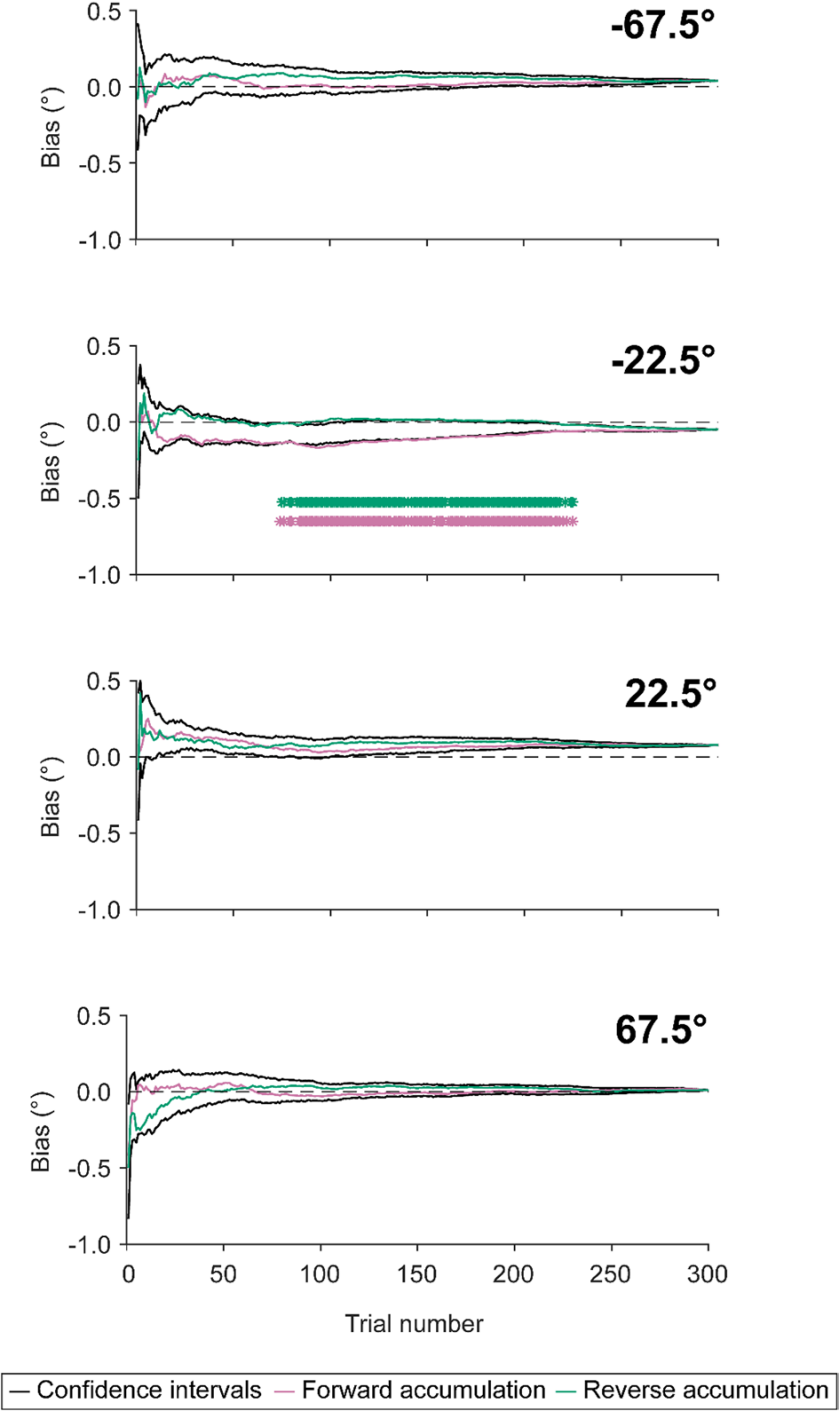
**Mean bias accumulation data for adaptation sessions** at each individual standard orientation (relative to the adaptor orientation), collapsing across adaptor conditions. Accumulated bias is calculated by taking the cumulative mean response spanning from trial 1 to 300. This was done in forward (i.e., ascending from 1; pink) and reverse (i.e., descending from 300; green) directions, however both are plotted in the same timeline to visualise differences in biases at the start vs. end of the adaptation session. Black lines represent 95% confidence intervals, calculated from null distributions obtained from permutation analyses. Asterisks represent instances where the mean bias accumulation falls outside of the confidence intervals, indicating statistically significant differences between forward vs. reverse accumulations at that particular time point. For accumulations collapsing across positive and negative standard orientations, see **Supplementary Figure S2**.

We were interested in measuring if participants’ response bias at the start of the session differs to their response bias at the end of the session. To investigate this, we ran the same sequential analyses as described above with trials in reverse order. In this case, we start with the last trial, working backwards to the first trial, which calculates the average response across all trials. We are then left with a separate bias accumulation trace for forward and reverse accumulations, plotting the cumulative bias across trials (Fig. 5; **pink and green lines**). Should participants display a different response bias at the start of the session as compared with the end, then the cumulative response biases should differ between the forward and reverse accumulations.

To formally quantify whether there was a significant difference between forward and reverse cumulative biases, we conducted permutation analyses. Such analysis acts to generate a null distribution with associated confidence intervals for each relative standard orientation condition^63^. In this case, the null hypothesis is that the direction of accumulation does not impact response biases. We ran 1000 permutations of the sequential analysis, using the trial-to-trial cumulative bias data from forward and reverse accumulations. To generate the null distribution, for a given permutation, we calculate an accumulation trace in the same manner as above. However, rather than performing a forward or reverse accumulation sweep across trials, on each trial the choice to perform the forward or reverse cumulative bias calculation is randomised. This is equivalent to creating a synthetic dataset in which there can be no effect of direction on accumulation. For example, the 10th trial may be randomly chosen to be forward-coded, in which case the cumulative response bias would be calculated from the first 10 trials participants completed. Conversely, if the 10th trial was randomly assigned to be reverse-coded, then the cumulative response bias would be calculated from the *last* 10 trials participants completed. The independent and random assignment of reverse- vs. forward-coding on each trial across permutations therefore yields a distribution of biases for a given trial under the null hypothesis of no directional differences. From these distributions for each trial attributed to each relative standard orientation condition, we calculate 95% confidence intervals by finding the.025th and.975th quantile of the permutation data for each trial (Fig. 5; **black lines**).

## Results

We quantified the extent to which prolonged adaptation to orientation-filtered videos influences subsequent orientation judgements. Participants completed three testing sessions in which we measured their baseline orientation bias, and then following adaptation to filtered video clips. The effects of adaptation were measured using a behavioural task in which participants judged whether a centrally presented test grating was tilted clockwise or counterclockwise relative to a peripherally presented oriented bar. The first session measured participants’ “baseline” orientation bias prior to viewing the adaptor stimulus. The second and third sessions were similar in design to a typical tilt-aftereffect experiment, where participants were shown clips of a film which had had its orientation information altered (i.e., the adaptor/top-up stimuli), which were separated by test grating judgement trials. Specifically, each participant completed a session with a cardinal (0° or 90°) adaptor, where the film had been filtered to have only one specified cardinal orientation present at relatively low spatial frequencies, and another session with an oblique adaptor (45° or 135°). The inclusion of a baseline session allowed us to assess each individual’s relative degree of adaptation to the adaptor stimuli. We assessed adaptation using a method of constant stimuli approach, calculating shifts in bias of participants’ clockwise/counterclockwise judgements relative to the orientation of the adaptor.

### Adaptation effects in response to naturalistic stimuli are minimal

We fit a GLMM to our response data using the offset between the test grating and standard orientation (i.e., ± 10°, ± 5°, ± 2.5°, and 0°) as well as the interaction between adaptor orientation (i.e., 0°, 45°, 90°, 135°, calculated relative to the baseline condition, which was also included as a predictor) and standard orientation (i.e., ± 22.5° and ± 67.5°) as predictors, with participants included as a random effect. The implementation of this GLMM is described in depth in Sect. Generalised linear multilevel modelling.

To assess the impact of adaptation on response biases, we first calculated observers’ baseline biases (Fig. 4A). Estimates associated with the baseline predictors in our GLMM suggested baseline biases were not statistically significant (see **Supplementary Table ****S1** for full model output). We subtracted these baseline biases from the bias associated with each corresponding adaptor condition, across adaptor orientations, thereby revealing response bias attributable to adaptation alone. We normalised standard orientations to represent their orientation relative to the *adaptor* orientation rather than to vertical (Fig. 4B).

The GLMM revealed only two instances where a combination of adaptor orientation and standard orientation (relative to the adaptor orientation) predictors were significant (see **Supplementary Table ****S1** for full model output). The first was a repulsive effect for the 0° adaptor orientation at the standard orientation of 22.5° (relative to the adaptor; *p* <.001). The second was an attractive effect for the 90° adaptor orientation at the standard orientation of 22.5° (relative to the adaptor; *p* =.042). Indeed, in the case of these two conditions, the strength of observed adaptation effects are much smaller than what we expected based on a classic tilt-aftereffect paradigm^8^. Further, these two conditions are greatly outnumbered by the 14 other conditions in which we observed no evidence of significant adaptation, paired with large variability in participants’ individual bias fits across conditions (Fig. 4D). Our results therefore suggest a broad lack of robust adaptation in response to our naturalistic movie stimuli. One possible explanation is that the embedding of adaptor stimuli in more naturalistic surrounds may act to reduce adaptation effects or requires longer exposure times to elicit strong adaptation effects. Alternatively, there may have been changes in adaptation throughout the testing sessions, mitigating the appearance of bias averaged across all trials, which we investigate below.

### Adaptation does not reliably change within a testing session

Beyond investigating the difference in response bias between baseline and the end of adaptation, we were further interested in how this response bias changes over time as participants undergo adaptation. We therefore investigated whether adaptation effects change over the course of a given session. In this section, we first outline the bias patterns observed in participants’ data. Following, we discuss the inferential approach taken to interpret the analysis’ results.

To investigate the time course of observed adaptation effects, we analysed the accumulation of participants’ response biases across time. The implementation of this analysis is described in depth in Sect. Exploratory sequential analyses. In brief, this was done by taking the cumulative average response from trial to trial to see how participants bias shifts over time (Fig. 5). To assess whether there is a difference in adaptation effects at the start vs. end of adaptation, accumulation analyses were run with responses in forward (Fig. 5, **pink lines**) and reverse order (Fig. 5, **green lines**). Separate analyses were conducted for each relative standard orientation to allow for consistent interpretation of whether a calculated bias is attractive or repulsive relative to the adaptor orientation. Comparing standard orientation conditions, there was noted variability in the time course of forward vs. reverse bias accumulation. For example, the 22.5° relative standard orientation condition displays greater consistency in forward vs. reverse traces than the − 22.5°.

We then tested for statistically significant deviations between forward vs. reverse accumulations in each relative standard orientation condition. To do this, we conducted permutation analyses on the time course data to generate a null distribution with associated 95% confidence intervals. The generation of confidence intervals allowed visualisation of when participants’ accumulated biases in forward and reverse directions are significantly different from one another (Fig. 5, **black lines**). We found significant deviation of response bias in the forward vs. reverse direction only when the relative standard orientation is −22.5° (Fig. 5, **second plot from the top**). In addition, we considered bias accumulations in the same manner for absolute standard orientations by reversing the signs of the bias for negative standard orientations, and combining these data with the corresponding positive standard orientation. Here, for both standard orientations, we found very few instances of differences in response biases for forward vs. reverse directions (see **Supplementary Figure S2** for visualisation). Inconsistent bias accumulation effects further suggests that embedding adaptor stimuli in more naturalistic surrounds in the manner of the current study may alter the nature of adaptation effects and/or may require longer exposure times to elicit.

## Discussion

We investigated visual adaptation to altered orientation statistics embedded in naturalistic stimuli. Participants watched films filtered to contain adaptors at a specific orientation and intermittently completed an orientation judgement task to measure shifts in response bias. We found very little evidence of adaptation across our conditions and, in the very few instances where significant adaptation was observed, effects were weaker than those observed in typical tilt-aftereffect studies. Indeed, we also found great participants to be highly variable in their response biases following adaptation. Further, we found adaptation effects do not consistently accumulate or fluctuate within a single testing session. Our results therefore suggest a divergence in the nature of adaptation between naturalistic movie viewing and typical adaptation studies. Of course, it is possible that the large divergence of the current paradigm from traditional adaptation investigations contributed to the lack of robust adaptation effects. However, the few small adaptation effects we did find are qualitatively similar to adaptation effects in response to isolated orientated contrast^6,7,9,10^. Additionally, evidence has been found for orientation adaptation aftereffects in response to windowed naturalistic image regions with strong orientation cues^64^. We therefore expect that strong adaptation effects would still emerge in the current results, should they be elicited.

### Naturalistic stimulus viewing elicits weak adaptation effects

Results of the current study suggest that adaptation to information embedded in naturalistic stimuli lacks robustness between adaptor orientations, as well as over the course of a single testing session. There are several elements of the current study that differ from typical adaptation investigations, which might contribute to the pattern of results found. First, the test stimulus in our experiment was relatively high contrast, which might act to limit adaptation effects^65^. However, tilt aftereffects have been demonstrated using high-contrast stimuli^53–55,66^. Furthermore, the reader can experience the effect themselves in simple high contrast demonstrations, like that shown in Fig. 1A. Hence, it is unlikely that such contrast-dependent adaptation could explain the current results.

Further, previous research has demonstrated clear adaptation effects within limited experimental sessions with as little as two minutes of adaptation^14^. However, unlike past experiments employing minimalistic stimuli, the current task inherently allows visual input to change substantially across adaptation due to the inclusion of eye movements in conjunction with a dynamic film stimulus. One possible explanation, therefore, is that the use of a dynamic stimulus, as in the current study, inherently weakens adaptation effects and/or requires longer exposure times to elicit to the same extent as in past research. It would be beneficial for future research to formally compare the use of minimalistic and naturalistic stimuli in adaptation studies, to elucidate key factors that may contribute to the observed effects.

### The impact of image phase

While the current study manipulated the oriented contrast distributions across film frames to have a single orientation at relatively low spatial frequencies, and equated orientation energy at all other spatial frequencies. However, we did not alter the phase information within each frame. Phase alignment, therefore, will have preserved the spatial structure of the film frames^62,67–69^. In the current study, stimulus processing still subjectively results in strong orientation cueing from structural information such as the edges of buildings, regardless of the manipulated oriented contrast energy. Indeed, previous investigations have successfully had participants match naturalistic images based on their spatial structures after undergoing manipulation of the overall phase alignment and being filtered to have uniform oriented contrast^67^. As such, the preservation of spatial structure in the current study’s stimuli is expected to have allowed meaningful engagement with the film, facilitating following of the storyline and appreciation of the scenes they were observing. It is therefore possible that such scene “understanding”, particularly in the current study where global structural information is in alignment with naturalistic image statistics, might have mitigated adaptation effects. This possibility is an exciting question for future research.

### The impact of participant’ stimulus viewing patterns

Given that participants freely viewed the video stimulus, it is likely that the peripheral visual field was inconsistently exposed to the adaptor stimulus, which is potentially relevant given the localised nature of adaptation effects^70^. Depending on a participant’s fixation distribution, parts of the visual field could be exposed to the adaptor stimulus while others would not. Further, it is possible that the areas that are exposed to the adaptor could align with the subsequent standard stimulus. This could be problematic due to the “El Grecco” effect, whereby a viewer who experiences a perceptual distortion for a given stimulus should experience that same distortion for their reproduction of that stimulus^71^. As such, their reproduction should reflect the “objective” nature of the stimulus, and not reflect the experienced distortion. In the current study, if the adapted area of the visual field was exposed to both the standard and test stimulus, both would be equally impacted by adaptation, preventing measurement of any adaptation effects that are present. However, such an issue would necessitate that participants consistently foveate particular peripheral areas of the visual stimulus during adaptation. Previous research has demonstrated viewers have a “central” bias, whereby they tend to fixate the centre of a display, even when viewing live-action film stimuli^59^. Additionally, visual exploration of naturalistic images is largely driven by salient low-level features that can change drastically in spatial location in dynamic film stimuli^72–75^. Further, if participants were to fixate particular peripheral areas of the film on every trial, we should not have observed any significant adaptation results. Hence, it is unlikely that such concerns could entirely explain the adaptation effects observed.

While it is unlikely that concerns regarding how participants engage with the visual stimulus can explain the observed results, future research could improve upon the current study by directly addressing such concerns. This could be done by having participants maintain fixation for the duration of the experimental session – however, this would reduce the naturalistic viewing conditions imposed in the current study and would likely require eye tracking to enforce. If eye tracking were implemented, participants could watch the film stimulus in a gaze-contingent manner, which would ensure that the visual field being adapted is controlled for and the standard stimulus could be positioned to account for this. Alternatively, experiments employing virtual/augmented reality (VR/AR) could be beneficial to explore these phenomena. However, the benefit of VR/AR would be to fully immerse participants in an environment with altered image statistics. This would likely be problematic in the case of measuring adaptation because it would be difficult to design an adaptation task with a standard that was not equally impacted by the adaptor.

### Adaptation to naturalistic stimuli is inconsistent

Overall, the current study suggests that adaptation is not robust in response to orientation statistics conveyed by live-action film stimuli as employed in the current study. Further investigation into the contribution of participants eye movements would elucidate the contribution of participants’ overt attentional deployment to the visual stimulus to observed adaptation results. In addition, the current study may point to longer exposure times being required to elicit adaptation effects in the context of more naturalistic viewing conditions. Future investigations reflecting the current results, however, would suggest strong adaptation effects in the literature to date might be a product of the abstract/simple stimuli that have been used.

## Supplementary Information

Below is the link to the electronic supplementary material.

Supplementary Material 1

## Data Availability

Behavioural data and GLMM code are available on the Open Science Framework: https:/osf.io/5evns/?view_only=ba38508be394490e85804691cd2f67e2.
