# Supplementary material for: Investigating orientation adaptation following naturalistic film viewing
Source: Sci Rep. 2025 Sep 26;15:33318. doi: 10.1038/s41598-025-21383-x (PMC12475178; doi:10.1038/s41598-025-21383-x)
Supplement: Supplementary file 1 — Supplementary Material 1 [file 41598_2025_21383_MOESM1_ESM.docx]

Investigating orientation adaptation following naturalistic film viewing

Emily J A-Izzeddin^1,2,3^*, Reuben Rideaux^3,4^, Jason B Mattingley^3,5^, William J Harrison^5,6^

^1^ Department of Experimental Psychology, Justus Liebig University Giessen, Giessen, Germany

^2^ Center for Mind, Brain and Behavior (CMBB), University of Marburg and Justus Liebig University Giessen

^3^ Queensland Brain Institute, The University of Queensland, St Lucia, Queensland 4072 Australia

^4^ School of Psychology, The University of Sydney, Camperdown, New South Wales 2006 Australia

^5^ School of Psychology, The University of Queensland, St Lucia, Queensland 4072 Australia

^6^ School of Health, University of the Sunshine Coast, Sippy Downs, Queensland 4556 Australia

* Corresponding author – Emily.A-Izzeddin@psychol.uni-giessen.de

**SUPPLEMENTAL MATERIALS**


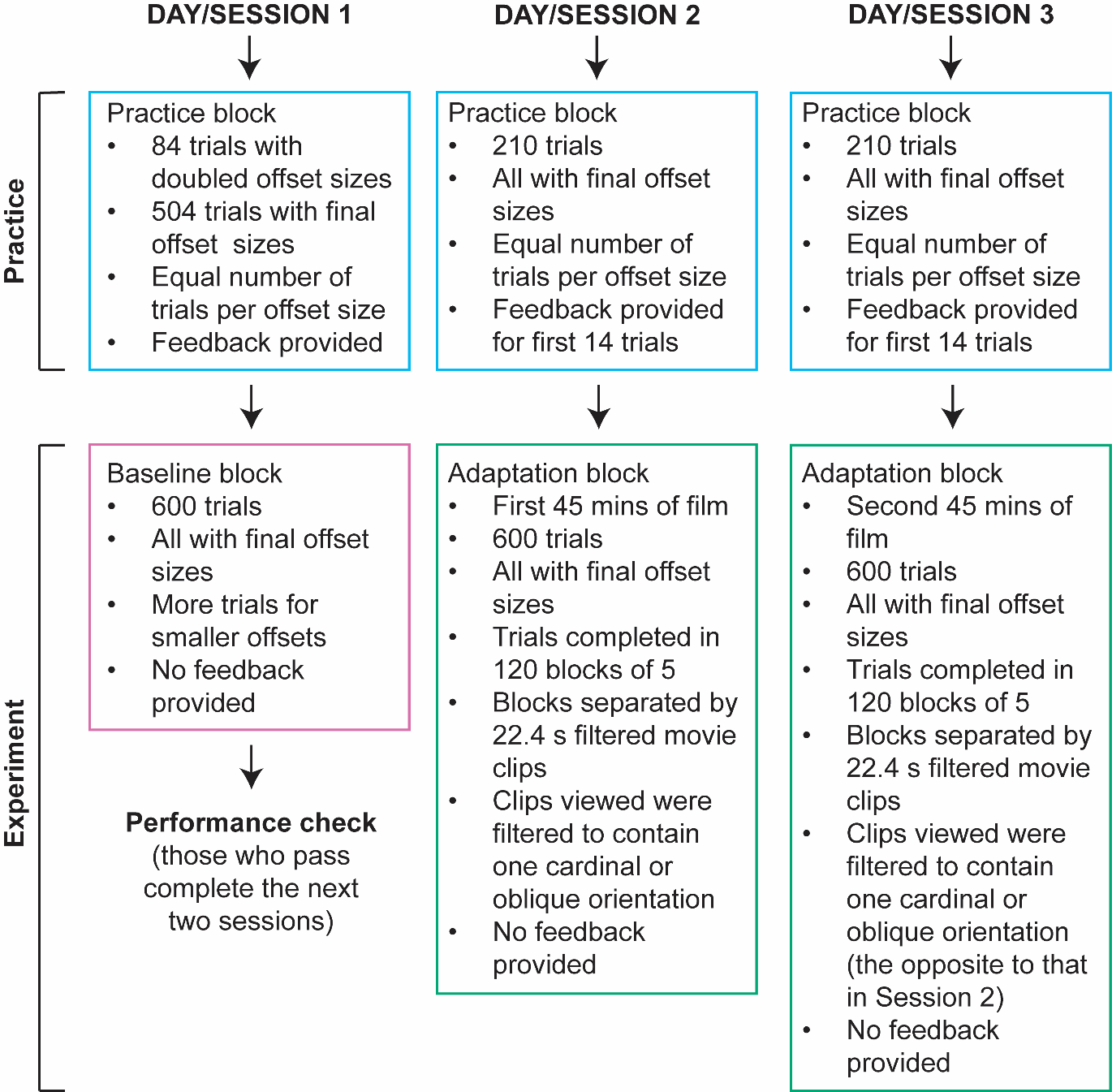


Figure S1. Overview of experimental sessions. Each participant completed an initial session (left column) that included a practice block of trials (blue box), followed by the baseline measurement (pink box). Participants’ baseline performance is assessed (see Practice and baseline testing in the main text). Participants who pass the baseline performance assessment go on to complete the next two sessions (middle and right column). Each of the final two sessions followed the same structure: participants initially did a block of practice trials (blue boxes), followed by an adaptation block (green boxes). In the adaptation blocks, participants performed the same behavioural task as in the baseline session. However, in the adaptation blocks, trials were completed in blocks of five, separated by a filtered movie clip, containing an adaptor that was either cardinal (0° or 90°) or oblique (45° or 135°). Each participant did one session with a cardinal adaptor and the other with an oblique adaptor. The order of cardinal vs oblique adaptors was counterbalanced across participants, as were combinations of the possible adaptor orientations.

Table S1: Full output for the GLMM defined by the equation: $\boldsymbol{y \sim}\boldsymbol{\beta}_{\boldsymbol{0}}\boldsymbol{+}\boldsymbol{\beta}_{\boldsymbol{1}}\boldsymbol{T}\boldsymbol{+}\boldsymbol{\beta}_{\boldsymbol{2}}\boldsymbol{F+}\boldsymbol{\beta}_{\boldsymbol{3}}\boldsymbol{S+}\boldsymbol{\beta}_{\boldsymbol{4}}\boldsymbol{FS}$. Here, $\boldsymbol{\beta}_{\boldsymbol{0}}$ is the intercept term, $\boldsymbol{\beta}_{\boldsymbol{1}}$ is the weight of the test stimulus offset relative to the standard, $\boldsymbol{T}$, $\boldsymbol{\beta}_{\boldsymbol{2}}$ is the weight of the adaptor condition, $\boldsymbol{F}$, $\boldsymbol{\beta}_{\boldsymbol{3}}$ is the weight of the standard orientation, $\boldsymbol{S}$, and $\boldsymbol{\beta}_{\boldsymbol{4}}$ is the weight of the adaptor/standard orientation interaction, $\boldsymbol{FS}$. Below, where we consider estimates attributed to combinations of F and S values, the S value has been adjusted to represent the standard orientation relative to the *adaptor* orientation (i.e., F). To partially pool coefficient estimates across participants, the GLMM included participant as a random effect.

| **Name** | **Estimate** | **SE** | **tStat** | **DF** | **pValue** |
| --- | --- | --- | --- | --- | --- |
| Intercept | 0.065 | 0.090 | 0.726 | 52587 | 0.468 |
| F_0 | -0.067 | 0.095 | -0.708 | 52587 | 0.479 |
| F_45 | 0.050 | 0.102 | 0.497 | 52587 | 0.620 |
| F_90 | -0.104 | 0.145 | -0.718 | 52587 | 0.473 |
| F_135 | 0.037 | 0.128 | 0.292 | 52587 | 0.771 |
| S_-22.5 | -0.414 | 0.136 | -3.048 | 52587 | 0.002* |
| S_22.5 | 0.090 | 0.170 | 0.531 | 52587 | 0.596 |
| S_67.5 | -0.115 | 0.129 | -0.892 | 52587 | 0.373 |
| T | -0.275 | 0.018 | -15.081 | 52587 | <.001** |
| F_0:S_22.5 | 0.567 | 0.130 | 4.350 | 52587 | <.001** |
| F_45:S_-22.5 | -0.191 | 0.141 | -1.355 | 52587 | 0.176 |
| F_90:S_-67.5 | -0.004 | 0.168 | -0.022 | 52587 | 0.983 |
| F_135:S_-22.5 | 0.099 | 0.164 | 0.604 | 52587 | 0.546 |
| F_0:S_-22.5 | -0.033 | 0.170 | -0.194 | 52587 | 0.846 |
| F_45:S_22.5 | 0.190 | 0.207 | 0.921 | 52587 | 0.357 |
| F_90:S_67.5 | 0.026 | 0.192 | 0.134 | 52587 | 0.894 |
| F_135:S_-67.5 | 0.064 | 0.131 | 0.491 | 52587 | 0.623 |
| F_0:S_-67.5 | -0.050 | 0.182 | -0.277 | 52587 | 0.782 |
| F_45:S_67.5 | -0.232 | 0.193 | -1.202 | 52587 | 0.229 |
| F_90:S_22.5 | 0.336 | 0.165 | 2.036 | 52587 | 0.042 |
| F_135:S_67.5 | -0.389 | 0.234 | -1.665 | 52587 | 0.096 |

* *p* < .005

** *p* < .001


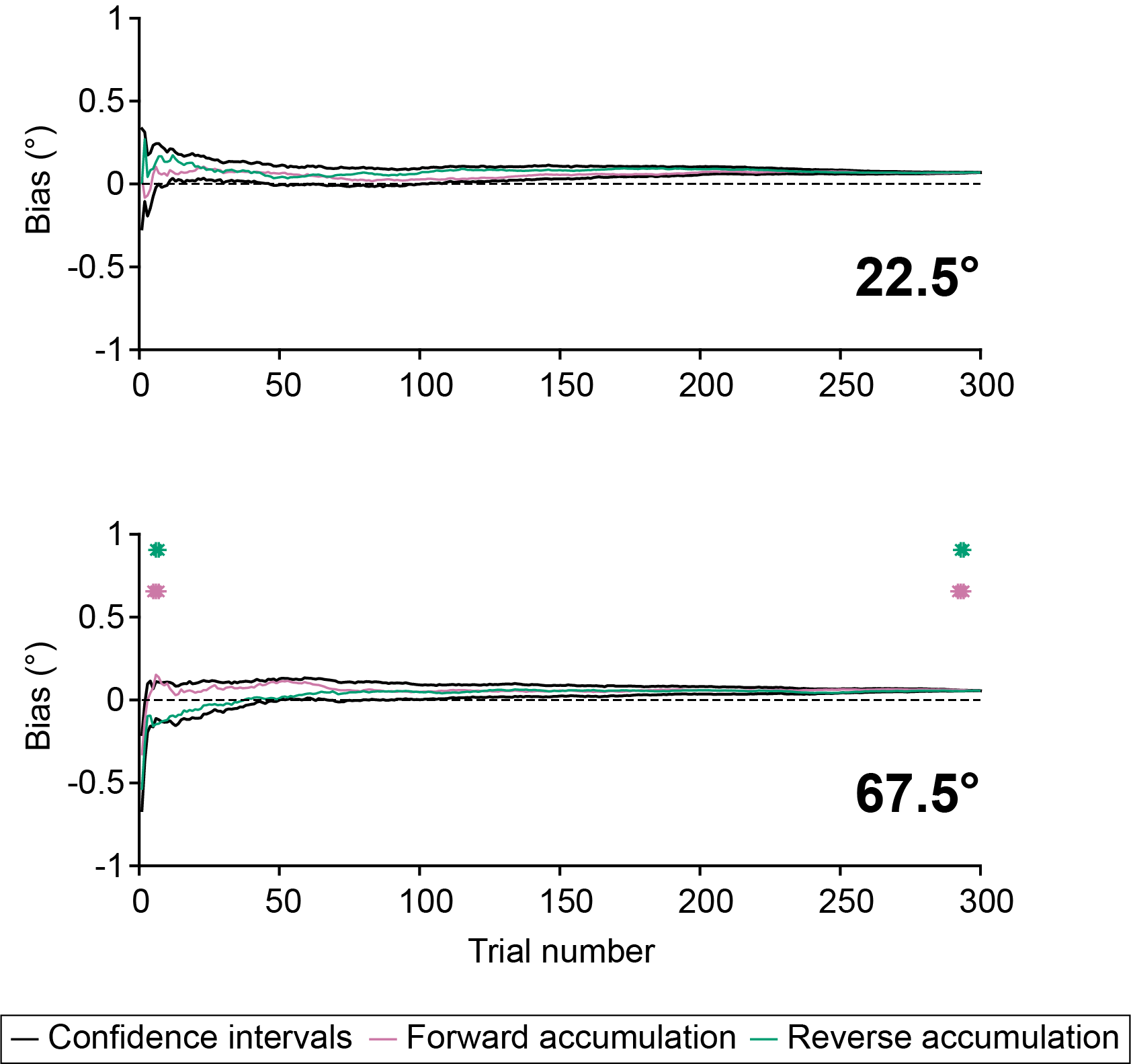


**Figure S2. Mean bias accumulation data for adaptation sessions for each absolute standard orientation (relative to the adaptor orientation), collapsing across adaptor conditions.** Accumulated bias is calculated by taking the cumulative mean response spanning from trial 1 to 300. This was done in forward (i.e., ascending from 1; pink) and reverse (i.e., descending from 300; green) directions, however both are plotted in the same timeline to visualise differences in biases at the start vs end of the adaptation session. To collapse across positive and negative standard orientations, we reverse the sign of the bias for the negative orientations and average with the corresponding positive standard orientation biases. Black lines represent 95% confidence intervals, calculated from null distributions obtained from permutation analyses. Asterisks represent instances where the mean bias accumulation falls outside of the confidence intervals, indicating statistically significant differences between forward vs reverse accumulations at that particular time point.
